# The State Medical Associations

**Published:** 1874-03

**Authors:** 


The Medical Association of the State of Alabama, convenes in Selma, on Monday the 13th day of April next. The South Carolina Medical Association meets next in Columbia, on Tuesday, the 14th of April.

The Medical Association of Georgia will assemble in Thomville on Wednesday the 1st day of April.

Let us urge upon our readers to lay aside, for a few days, their busy avocations and meet the brethren of their respective States in fraternal council and social enjoyment. These annual re-unions are certainly productive of laudable professional emulation, as well as instructive by the comparison of individual views and observations with the opinions and experiences of our fellows. The relaxation from the tread-mill of our daily routine of professional duty is re-invigorating to the mind and body, whilst the social amenities, which usually attend upon these annual gatherings, are in a high degree promotive of that cordial good-will and *bonhomie* which ought ever to characterize the members of a great and learned profession. Let no one miss the Association who can possibly go.

